# Workplace learning

**DOI:** 10.1007/s40037-012-0005-4

**Published:** 2012-02-07

**Authors:** Tim Dornan

**Affiliations:** Department of Educational Development and Research, Maastricht University, PO Box 616, 6200 MD Maastricht, The Netherlands

**Keywords:** Workplace learning, Undergraduate medical education, Residency, Continuing medical education, Qualitative research

## Abstract

This critical review found Dutch research to be strong at the undergraduate and residency levels and more or less absent in continuing medical education. It confirms the importance of coaching medical students, giving constructive feedback, and ensuring practice environments are conducive to learning though it has proved hard to improve them. Residents learn primarily from experiences encountered in the course of clinical work but the fine balance between delivering clinical services and learning can easily be upset by work pressure. More intervention studies are needed. Qualitative research designs need to be more methodologically sophisticated and use a wider range of data sources including direct observation, audio-diaries, and text analysis. Areas for improvement are clear but achieving results will require persistence and patience.

## Introduction

Workplace learning is as old as medicine itself. Before the Flexner report of 1910 [1], the term tended to mean working for more senior doctors who were accountable to nobody for the quality of their work, which could be very poor. Flexner was commissioned to write his report to improve that state of affairs. One result of the report was that medical education came to be delivered (or at least supervised) by universities. Another was that educational standards rose. Teachers were now academic practitioners but the tradition of learning through service persisted. Other articles in this issue describe newer developments in medical education—in student selection, faculty development, assessment, simulation, and competency development. Old-fashioned workplace learning, however, remains important because practice has to be learned by practising. Workplace learning exists in medical curricula in many different guises: Early clinical experience, clerkships, residency, and continuing medical education. I start with some personal observations on how workplace medical education is practised in Dutch-speaking Europe and then review Dutch education research in the field. Throughout the article, I use the adjective ‘Dutch’ to refer to medical education in Flanders as well as the Netherlands.

## The practice of workplace learning

I am struck by differences between my Dutch experiences and what I have seen in Britain and some other parts of the world. Dutch medical students—like ones in North America—have rich workplace learning opportunities. Particularly in the senior clerkship years, they participate in practice in a way that is rarely seen in contemporary Britain. Work I did with Maastricht University and the Open University of the Netherlands showed that participation is central to students’ identity development, and identity development is at the centre of their learning [2]. So there is much to commend contemporary Dutch undergraduate workplace learning. But there is a potential cost to patients. de Feijter et al. [3] showed how learning from participation can confront medical students with difficult choices. A nurse might, for example, ask a student to do something they are not fully trained or authorised to do. On the one hand, they should refuse for the sake of patient safety. But on the other, their identity formation is strongly linked to performing tasks, so there is an incentive to perform the unsafe act. That tension could be a usefully formative one in a well-supported learning environment but there is a fine balance between providing too much and too little support. The fact that such a tension emerged in recent Dutch research suggests the balance may not always be achieved. Much UK health care is delivered by the National Health Service and patient safety is such a politically sensitive issue that the tension described by de Feijter et al. [3] is much less apparent in contemporary Britain (though I was wholly familiar with it 30 years ago) because students now have such limited opportunities to participate in practice.

A very positive feature of Dutch medical education is how the transition [4] between senior medical student and qualified doctor has been deliberately blurred. Dutch medical students are progressively exposed to the tension of practice, which may tip the balance towards a more favourable transition [4]. Contemporary Dutch undergraduate curriculum design is a nice example of what Kennedy and colleagues termed ‘progressive independence’ [5]. For the reasons given above, the trend in the UK has been in just the opposite direction, though efforts are now being made to reverse it. Research in Manchester showed how the abruptness of newly qualified UK doctors’ entry to practice can compromise patient safety by contributing to prescribing errors [6]. That is a counterpart to the situations analyzed by de Feijter et al. [3]. It shows just how important it is to achieve safe, legal, progressive immersion in practice. Despite the concerns I expressed in the previous paragraph, I think Dutch progressive immersion is a better approach to workplace learning than the UK’s relative exclusion of medical students from practice until the moment of qualification, followed by abrupt immersion.

My international comparison is less favourable towards the Netherlands and Flanders when I view the period after qualification. Newly qualified UK doctors complete a 2-year rotation through different specialities, enter a common core rotation (e.g. surgery or internal medicine), and then specialize (e.g. neurology, rheumatology). Medical graduates in the Dutch-speaking world do not have a period of general professional education so they develop their CanMEDS competencies [7] within a speciality-specific milieu. Even if there is more participation in practice in Dutch than UK undergraduate medical education, I doubt it can fully provide the insights into other fields of practice that are so essential to interdisciplinary collaboration. I do, however, have a positive observation about Dutch postgraduate education to offset that negative one. Boor et al. [8–10] demonstrated that a widely used measure of postgraduate education environments lacked validity evidence, teased out the dimensions of the learning environment construct, then developed and validated the D-RECT instrument, whose 11-subscale structure makes it a powerful tool for formative and summative evaluation and quality development. They have made a valuable contribution to international scholarship by developing a valid means of measuring the quality of postgraduate learning environments.

Finally, a reflection on continuing professional development (CPD; or continuing medical education, CME). It is a very prominent part of the medical education continuum in Britain, the USA, Canada, and Australia, which does not seem so much the case in the Netherlands and Flanders. But is that all bad? I have argued elsewhere that the UK discourse of CPD is a disempowering, regulatory discourse rather than a discourse that empowers lifelong workplace learning [11]. CPD is an important topic, because it concerns maintaining the quality of expert professional practice. It is under-researched compared with other aspects of medical education and presents good opportunities for education research that can impact on the quality of health care.

## Research into workplace learning

### Medical student education

I use a piece of my own research, a realist synthesis [12] of how medical students learn in workplaces [13], to show how Dutch publications have contributed to the scholarship of workplace learning. Our team, which includes two Dutch researchers, six others, and myself identified papers published between 2000 and 2006 that form an evidence-base of how medical students learn in workplaces. Forty-seven percent of the 168 papers originated from the USA, 19% from Britain, 11% from mainland Europe and the Nordic countries, 7% from Canada, 9% from Australia or New Zealand, and 7% from other parts of the world. Looking more closely at the Dutch contribution, 14 papers (8%) were conducted solely in the Netherlands or Belgium, or had Dutch institutions as collaborators. After excluding the four papers of my own which were co-published with Maastricht University but conducted on British soil, 10 Dutch contributions remained [14–23]. Fieldwork was done at the VU University, Amsterdam in four studies [15, 17, 18], at Maastricht University in three studies [16, 19, 20], and at the Erasmus University, Rotterdam [21], Catholic University of Leuven [22], and the University of Antwerp [14] (one study each). Eight studies concerned clerkship learning [14–18, 21–23], one concerned the transition to clerkship [20], and one reported a longitudinal experience in primary care during the early curriculum years [19]. Five of the clerkship studies were purely observational and three had an element of intervention [18, 19, 23]. Nine were purely qualitative or used mixed methods whilst one used structural equations modelling to analyze numerical data [21].

The findings of the Dutch studies were quite consistent with one another and similar to findings in other countries. The two main determinants of learning during clerkships were the quality of supervision and casemix [16, 21]. Better supervision could influence and compensate for limited casemix [21]. Supervision directly enhanced academic performance [21]. Feedback was most effective when given by someone who knew the student and whom the student knew [17]. Sympathetic and warm feedback had important positive effects on students’ emotions and harsh or absent feedback had negative effects [17, 20]. Learning environments that were more orientated towards education (rather than pure service provision) were motivating, whereas learning environments in which education was not a priority left students feeling abandoned [22]. Students did not always receive high-quality supervision and feedback [15, 17, 18, 20]. When given, feedback was not always based on observation of their performance [17]. The ‘learning by trial and error’ [15] that resulted left students in doubt about their proficiency and whether they were attaining curriculum objectives. Being given clear learning outcomes [19] and being coached in clinical skills [14] helped students learn. The three studies that had an interventional component are very informative in that the interventions made little difference. The introduction of an in-training assessment scheme had little, if any, effect on supervision and feedback because residents were unclear about their roles and students were reluctant to reveal their weaknesses to their assessors [23]. Attempts to improve the quality of supervision and feedback in a surgical clerkship had a limited impact on students’ hit and miss exposure to relevant casemix and the supervisory support to their learning [17, 18].

The fieldwork on which the findings in the previous paragraph are based is now somewhat out of date so they may not reflect what is happening on the ground today. I suspect, however, they do. Changing the ‘tea-steeping’ model (blocks of experiential learning by immersion within functioning clinical units) to a more outcome-focused, structured, instructed, and supervised model means overcoming a lot of inertia, as discussed in Cooke and colleagues’ Flexner centenary monograph [24]. Likewise, a recent review concluded that constructive feedback based on personal knowledge of students is generally absent in workplaces [25]. So, the findings of our review ring true despite their age.

I have looked for Dutch lines of inquiry into undergraduate medical education since 2006. My (doubtless incomplete) scan identified several. One, conducted in general practice, explored the consequences of placing learners in supportive environments [26, 27]. It is well established that two important dimensions of workplace instructional quality are high-quality supervision and access to appropriate patients. Promotion of independence, this research showed, is an important third dimension. High-quality supervision helps students learn independently from the casemix they have access to [27]. The same authors explored medical students’ learning in primary care from a sociocultural perspective and found that students form their professional identities within a private ‘developmental space’ under the combined influence of their workplace context, personal interactions, and professional ones [26].

Contemporary research into communication education again shows inertia. Communication skills training—mostly provided in the pre-clerkship years—aims to equip students with tools for patient-centred practice. Bombeke et al. [28] found exposure to hospital environments in the clerkship years counteracted patient-centred orientations developed in the earlier years. Lack of student self-efficacy, pressures of working environments and negative role models contributed to this decline of patient-centredness. A lack of patient-centred, self-caring, and self-aware role models in clerkship learning environments, their research suggests, may be responsible. The findings of a second study by the same researchers, which compared students who had received communication skills training with students who had not, were really rather alarming [29]. Students trained in communication skills showed a greater decline in patient-centredness during clerkships than students who had not been trained in communication skills. Communication skills training, the study suggested, may accentuate the clash between student idealism and workplace reality, which led to a decline in patient-centredness. Contemporary medical practice, it seems, is not patient-centred enough to serve as an educational model. One wonders, then, how it will ever be possible to make doctors more patient-centred. The study certainly suggests that communication skills education confined to the early curriculum years will not do the trick.

A third cluster of recent studies, from Groningen, concerned transition from pre-clerkship to clerkship education [30], the influence of learning environments, [31, 32] and how students learned within them [33, 34]. van Hell et al. [31, 32] found that feedback was most valued by students when it came from a doctor rather than an allied professional, was based on direct observation of their behaviour, and/or was initiated by themselves. Students’ ratings of the value of learning environments were higher when they spent more time in them and were more active participants [32]. Clerkship students used diverse learning strategies [33] and were motivated by comparing themselves with higher performing members of their peer group [34].

### Residency

I recently searched the international literature for empirical research into how residents learn. Remarkably little has been published. I judged two lines of enquiry to be particularly informative. Both were qualitative and both were Dutch [35, 36]. Residents’ learning, according to those papers, always starts from experiences encountered in the course of clinical work [35, 37], although the sheer pressure of clinical workload can easily reduce the value of workplace learning [36]. So, residents’ most important learning is ‘informal’ [36], as has been shown in other professions [38]. One of those two studies [36] was into how residents learn from deliberate practice while the other [35] explored how residents gave personal meaning to their workplace experiences, supported by their supervisors [37]. Teunissen et al. continued their research into personal meaning with two further studies. One was an experiment, which showed how ‘priming’ junior residents with an extraneous line of thought influenced their germane thinking about clinical problems [39]. This experiment supported their theory that residents’ interpretations of workplace experiences are influenced by personal knowledge and showed that extraneous factors have a stronger influence in junior than senior residents [39]. A second study by the same group evaluated two ‘dispositions’ of trainees and how they related to one another: One was being disposed to learn versus being disposed to make a good impression on others. The other disposition was towards seeking or not seeking feedback, given its perceived benefits and costs to the resident. The paper makes two important points: One is that residents are not passive recipients of feedback; feedback is an active discourse between supervisor and supervisee. The second point is that specialists’ style of giving feedback influences residents’ learning. Supportive specialists give feedback in a way that helps residents perceive more benefits and fewer costs [40].

Returning to my international review of research into how residents learn, one of the four remaining papers—which contributed consensus data about important factors in workplace learning environments—was Dutch [41]. The remaining three non-Dutch papers examined factors that influence residents’ participatory learning [42], the exchange of tacit knowledge between anaesthesiologists [43], and tensions between patient care and learning [44].

## Conclusion

According to this survey, the Dutch contribution to international scholarship in workplace learning is strong at the undergraduate and residency levels and absent at the CME level. A positive feature of the Dutch effort is the amount of high-quality research into residency education. A methodological weakness of the workplace learning research I have reviewed—in common research from other countries—is an excess of observational over interventional/experimental research. Qualitative workplace research tends towards focus groups and interviews in which researchers take respondents’ words as truth, rather than being critical about why respondents say the things they do in the research context. There are qualitative methodologies that address those concerns. The analytical heuristics of phenomenology and discourse analysis, for example, address that epistemological problem. Workplace learning research could benefit from alternative methods of data collection: Direct (participant) observation and audio-diary techniques, for example, give near contemporaneous accounts of learning, which reduce the problem of respondents’ experiences being reconstructed in retrospect to fit the research. Even without using phenomenology or discourse analysis, analytical approaches could be more sophisticated. Grounded theory had a strong influence on the early years of qualitative research, leaving a legacy of work that starts from no identified theoretical position and never reaches one. Grounded theory has a clear place, particularly in ‘scoping’ a field of research, when it generates new theory. ‘Thematic analysis’, in my view, has a more limited place, because it too rarely states its epistemological position and too often assumes that some objective truth resides within research respondents’ spoken words. Constructivist grounded theory is showing promise as a methodology that addresses some of the concerns expressed above. By using prior theory to provide ‘sensitizing insights’ that can be applied to data interpretation, it allows new theory to be built on pre-existing theory. There are examples of this in the Dutch work I have reviewed. Teunissen et al. [35], for example, allowed one grounded theory study to inform a second one [37], and then elaborated their theory programmatically by means of well-theorised experimental [39] and quantitative survey research [40]. Bombeke et al. derived sensitizing concepts from an ‘Attitude-Social influence-Self efficacy model’ and used them to analyze their patient-centredness data. de Feijter et al. [3] used Activity Theory in an informative way to reveal tensions in patient safety education while van der Zwet et al. concept of ‘developmental space’ was informed by sociocultural learning theory [26].

So what, finally, can we conclude about the state of the art in workplace learning? Workplaces afford rich learning opportunities, which are integral to their primary role—getting jobs done—but in constant tension with it. That tension is responsible for both the greatest successes and the greatest failings of workplace learning. Learning is mediated by the relationships that exist between learners, peers, more experienced practitioners, other health professionals, and patients. Participation in the activities of workplaces is a discourse, in which all participants play active parts. Supervision, feedback, and other teaching and learning activities are, likewise, discourses in which learners play important parts. Each workplace has its own rich cultural history, which means they respond slowly to efforts to change them. Humanistic qualities of practitioners, which have not traditionally been given the importance they have now assumed, are the essential ingredient of effective workplace learning environments. Education research has given clear direction about how those environments can be improved, but improving them will require persistence and patience.

## Essentials

- Conscious effort is needed to make working environments conducive to learning as well as ‘getting the job done’.
- Constructive feedback from a supportive practitioner who is known to a learner aids learning.
- Excessive workload makes it hard for residents to learn from practice.
- Continuing education is a phase of the lifelong learning continuum that tends to be neglected.
- There is more to qualitative research than transcribing what people say in interviews or group discussions and analysing it thematically.
